# Multi-Modal Haptic Feedback for Grip Force Reduction in Robotic Surgery

**DOI:** 10.1038/s41598-019-40821-1

**Published:** 2019-03-21

**Authors:** Ahmad Abiri, Jake Pensa, Anna Tao, Ji Ma, Yen-Yi Juo, Syed J. Askari, James Bisley, Jacob Rosen, Erik P. Dutson, Warren S. Grundfest

**Affiliations:** 1 UCLA Center for Advanced Surgical and Interventional Technology (CASIT), Los Angeles, USA; 2 UCLA Henry Samueli School of Engineering and Applied Science, Los Angeles, USA; 3 UCLA Department of Surgery, Los Angeles, USA; 4 UCLA Department of Neurobiology, Los Angeles, USA

## Abstract

Minimally invasive robotic surgery allows for many advantages over traditional surgical procedures, but the loss of force feedback combined with a potential for strong grasping forces can result in excessive tissue damage. Single modality haptic feedback systems have been designed and tested in an attempt to diminish grasping forces, but the results still fall short of natural performance. A multi-modal pneumatic feedback system was designed to allow for tactile, kinesthetic, and vibrotactile feedback, with the aims of more closely imitating natural touch and further improving the effectiveness of HFS in robotic surgical applications and tasks such as tissue grasping and manipulation. Testing of the multi-modal system yielded very promising results with an average force reduction of nearly 50% between the no feedback and hybrid (tactile and kinesthetic) trials (p < 1.0E-16). The multi-modal system demonstrated an increased reduction over single modality feedback solutions and indicated that the system can help users achieve average grip forces closer to those normally possible with the human hand.

## Introduction

Since the introduction of the first robotic surgical systems in 1985, the field of surgical robotics has gone through many changes with newer systems demonstrating increased ease of operations as well as decreased operative times compared with typical laparoscopic surgery^1^. Benefits of robotic surgery over conventional laparoscopic surgery include motion scaling for finer motion control, stereoscopic vision, increased dexterity, and additional degrees of freedom in motion^2–6^. However, these benefits have also come at a cost. Compared to conventional open surgery and even laparoscopic surgery, robotic procedures suffer from a complete loss of haptic feedback. The loss of haptic feedback coupled with the inherent ability of robotic surgical systems to apply strong compressive and shear forces, have led to increased risk of tissue damage, reduced performance, and increased number of mistakes^7–10^. Ultimately, these risks translate to clinical outcomes, meaning, greater tissue damage, more pain, and longer recovery times for the patient^11–14^. As robotic surgery gains popularity, it becomes imperative that haptic feedback systems (HFS) become a standard feature of commercially available surgical robotic systems.

In the past decade, there have been significant changes to the field of haptics and numerous haptic feedback solutions have been investigated for surgical robotics^13,15–19^. More primitive force feedback implementations have been included as part of some of the more modern surgical robotic systems^17,20^. Kinesthetic force feedback (KFF) has been perhaps the most comprehensively researched area of haptics^21–25^ because of its relative ease of integration with the master controls of surgical robots. However, haptics is not limited to kinesthetic force feedback. In fact, the sense of touch in humans involves the synergistic activation of mechanoreceptors both in the skin (i.e. tactile feedback) and in the muscles (i.e. kinesthetic force feedback)^23^. Some research groups have focused on tactile feedback as an alternative to force feedback, targeting activation of mechanoreceptors in the fingertips through pneumatic tactile feedback^26^ and/or vibrotactile feedback^27–29^. Studies have shown that these feedback solutions can significantly impact the performance of surgeons during the procedure^30–32^ and reduce tissue damage^10,12,16,33^. More recently, some systems have attempted development of multi-modal HFS by coupling thermal feedback with tactile feedback^34,35^. However, no significant investigations have been conducted to evaluate impact of such solutions in MIS applications.

Despite extensive research in the field of haptics, current feedback technologies are far from replicating the natural sense of touch that a surgeon receives in conventional open and even laparoscopic surgery. One reason for this is that none of these systems attempted to restore the synergistic behavior that takes place when various mechanoreceptors in the skin and muscles simultaneously become activated in real touch. In fact, nearly all haptic feedback systems have targeted only a single modality of feedback. The perception of feedback is therefore hindered because the brain does not receive signals from all the sensory pathways normally involved in the sense of touch.

A multi-modal haptic feedback system refers to a HFS that seeks to sense and convey more than one aspect of touch. By targeting multiple classes of mechanoreceptors in the skin and muscles, and conveying both tactile and proprioceptive information, multi-modal HFS engenders the promise of a more natural and complete HFS, one that hopes to bring haptics one step closer to real touch. Due to the engineering challenges that arise from attempting to integrate multiple sensing and feedback modalities while still maintaining small footprints necessary for application in surgery, multi-modal HFS has not been extensively studied. In more recent years, some attempts have been made on the development of bi-modal haptic feedback systems for surgical applications^15,33,36–38^, however, conclusive outcomes have not yet been achieved.

The use of haptic feedback for reduction of tissue damage in robotic tasks involving average forces generated during grasping and manipulation of soft tissue has previously been extensively studied^26^. Despite evidence that both tactile and kinesthetic feedback are effective at reducing forces applied to the tissue^12,39–41^, the effects of combining both tactile and kinesthetic feedback into one system have not been evaluated^11^. We hypothesize that a multi-modal HFS, which can target both mechanoreceptors in the skin and the muscles and provide simultaneous tactile and kinesthetic force feedback, can be more effective in reducing excessive grip forces during robotic surgical tasks.

## Results

**Figure 1:**
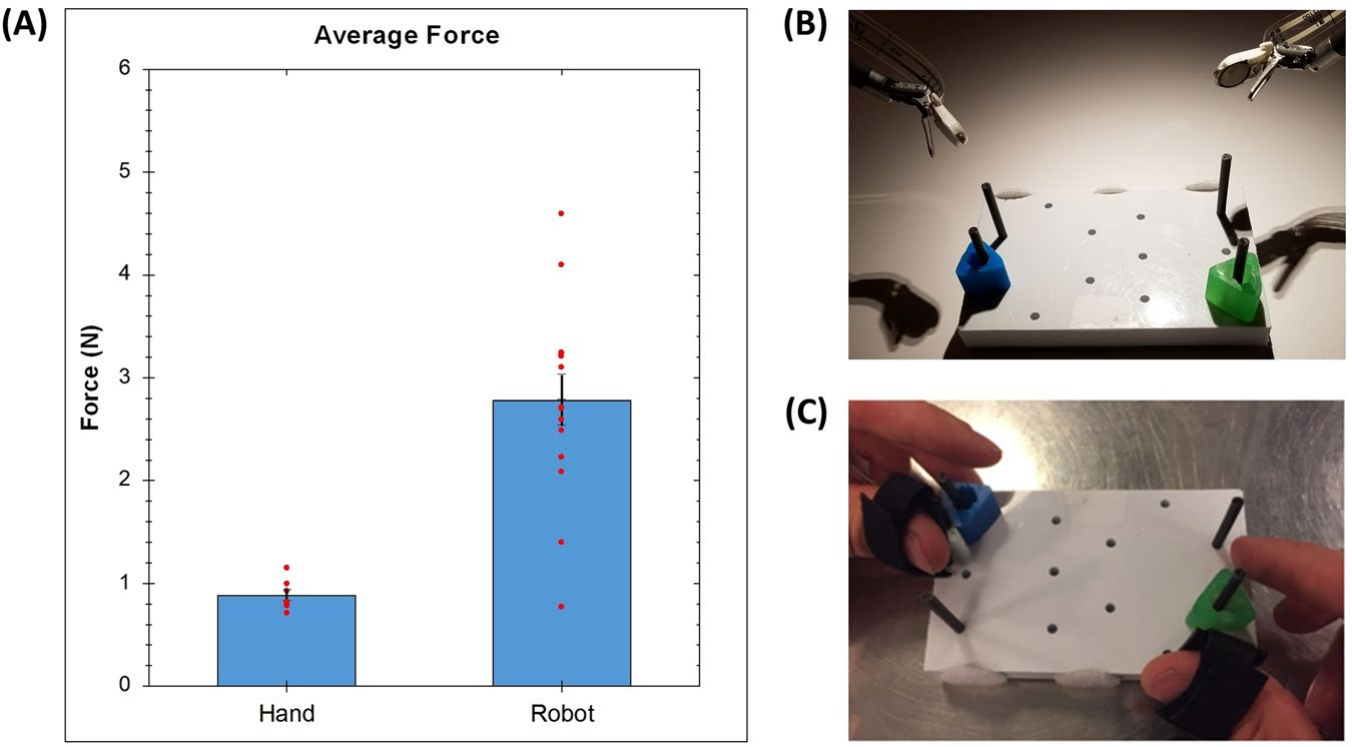
Comparison of Average Grip Force during a peg-transfer task. Task performed using human hands and when performed using da Vinci robotic surgical system without HFS: (**A**) Data from a 6-subject two-handed peg transfer performed using human hands is compared against data from the no feedback trial in the hybrid HFS study (Figs 2A and 3). (**B**) Two-handed peg transfer using the da Vinci IS1200 robotic surgical system. (**C**) Two-handed peg-transfer performed using human hands with force sensors installed on one finger.

**Figure 2:**
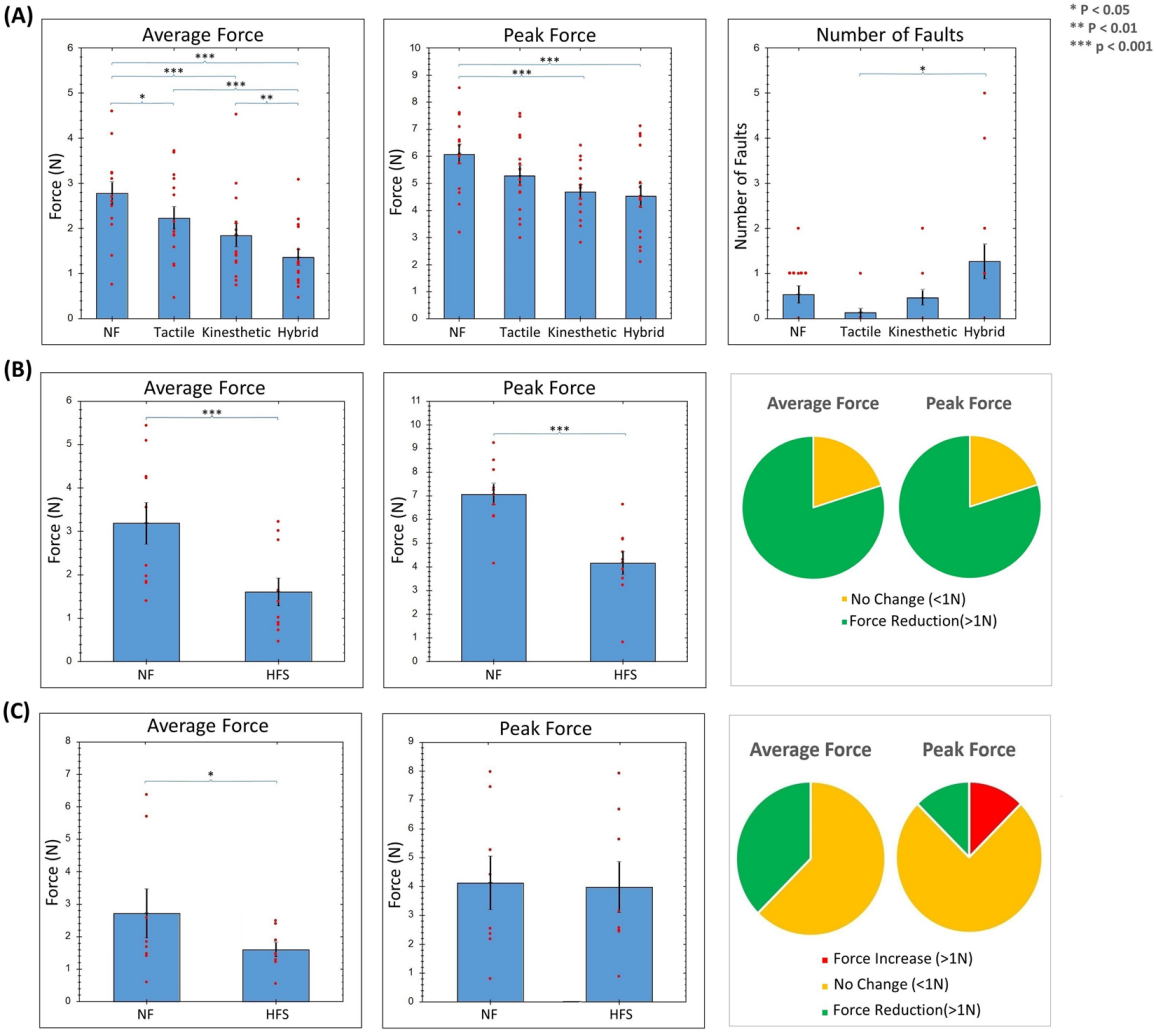
Evaluation of performance under different feedback conditions. (**A**) Comparison of Average Grip-Force, Peak Grip Force, and Number of Faults under different feedback conditions when utilizing the da Vinci IS1200 for peg transfer tasks. (**B**) Comparison of Average Grip Force and Peak Grip Force between No Feedback and Kinesthetic-Tactile Feedback condition during a porcine bowel run in novice subjects. Pie chart showing the inter-subject variation in this study. (**C**) Comparison of Average Grip Force and Peak Grip Force between No Feedback and Kinesthetic-Tactile Feedback condition during a porcine bowel run in expert subjects. Pie chart showing the inter-subject variation in this study.

The goal of haptic feedback systems is to restore the sense of touch available when performing tasks with human hands. Figure 1 shows the results of a benchmark study, in which the average grip force when 6 subjects performed two-handed peg-transfers with their own hands, was compared to when 15 subjects performed the task without any feedback on the da Vinci robot (*data from* Fig. 2A). These data showed that average grip force when performing a peg-transfer with a human hand was a mere 0.88N with a standard deviation of 0.15N. On the other hand, the same task performed using the da Vinci system without HFS, resulted in grip forces averaging 2.78N (std. dev. 0.96N).

### Bi-Modal Pneumatic Kinesthetic-Tactile HFS

The results from the experiments conducted using the pneumatic variation of the kinesthetic force feedback system show (Fig. 2A) that the average grip force is significantly lower (ANOVA^42^, p-value (p) = 2.4E-9, Degrees of freedom (Df) = 3, F-statistic (F) = 24.59) when feedback is provided. Compared to the no feedback condition, average forces were lower when tactile feedback (Tukey^43^, p = 0.017), kinesthetic feedback (Tukey^43^, p = 1.66E-6) or hybrid feedback (Tukey^43^, p < 1.0E-16) are provided. The bi-modal kinesthetic-tactile HFS also performs better than both tactile-only (Tukey^43^, p = 5.64E-8) and kinesthetic-only (Tukey^43^, p = 0.0027) feedback conditions.

With regards to the peak grip force, significant difference was also observed between the groups (ANOVA^42^, p = 0.0007, Df = 3, F = 6.876). While no significant improvement can be seen between the tactile feedback and the No Feedback condition, both kinesthetic (Tukey^43^, p = 0.0008) and hybrid HFS (Tukey^43^, p = 0.0001) conditions display a significant reduction in peak grip force compared to when no feedback was present.

The number of faults (Fig. 2A), i.e. the number of times the subject dropped the peg, appears to be significantly different among the groups as well (ANOVA^42^, p = 0.005, Df = 3, Chi-Square Statistic (χ^2^) = 12.702). While there are no significant differences between no feedback and the feedback conditions, a higher number of faults is observed when hybrid HFS was activated, compared to the tactile feedback group (Tukey^43^, p = 0.012).

The results from the peg transfer studies show the clear benefits of providing haptic feedback in reducing grip force. All feedback modalities performed significantly better than the no feedback condition. Most importantly, there is also a clear indication that the multi-modal kinesthetic-tactile feedback system is significantly better than both single-modality feedback solutions, benefiting from the synergistic relationship from the activation of mechanoreceptors in the skin and muscle. This leads to a more natural sense of touch, allowing the subjects utilizing multi-modal HFS to apply forces nearly 50% less than when no feedback is provided.

### *Ex-Vivo* Evaluation of Bi-Modal Kinesthetic-Tactile HFS

The *ex-vivo* bowel run experiments were designed with the goal of allowing comparison against the single modality tactile feedback system demonstrated in our previous work^26^. The results of the *ex-vivo* study in novice subjects (Fig. 2B) show a significant reduction in average grip force (T-test^44^, p = 0.00025, Df = 9, T-Statistic (T) = 5.827) and peak grip force (T-test, p = 0.00024, Df = 9, T = 5.861) in the hand that received feedback from the bi-modal HFS.

Looking at the inter-subject variation (Fig. 2B), it can be clearly seen that with regards to both average and peak grip-force, nearly all novice subjects benefited from the presence of the bi-modal kinesthetic-tactile HFS. These results clearly highlight the advantage of the newly developed multi-modal HFS over the traditional single-modality implementations^26^. While these previous studies showed large inter-subject variation and limited conclusive evidence as to the benefits of tactile feedback, the results of the *ex-vivo* bowel run (Fig. 2B) clearly indicate the consistent benefits provided by the multi-modal HFS.^26^. While these previous studies showed large inter-subject variation and limited conclusive evidence as to the benefits of tactile feedback, the results of the *ex-vivo* bowel run (B) clearly indicate the consistent benefits provided by the multi-modal HFS.

In expert surgeons, the same trend followed (Fig. 2C) with the hand receiving feedback showing a significant reduction in average grip force (Wilcoxon^45^, p = 0.0234, V = 2). No significant difference was observed in peak grip force (T-test^44^, p = 0.677, Df = 7, T = 0.434) and the number of faults for expert surgeons. Looking at the inter-subject variation, most subjects benefited from the presence of HFS, though the impact was less apparent than in novices.

The effectiveness of the bi-modal kinesthetic-tactile HFS warranted further study of the multi-modal HFS, utilizing vibration to help reduce excessive peak grip forces.

### Tri-Modal HFS

**Figure 3:**
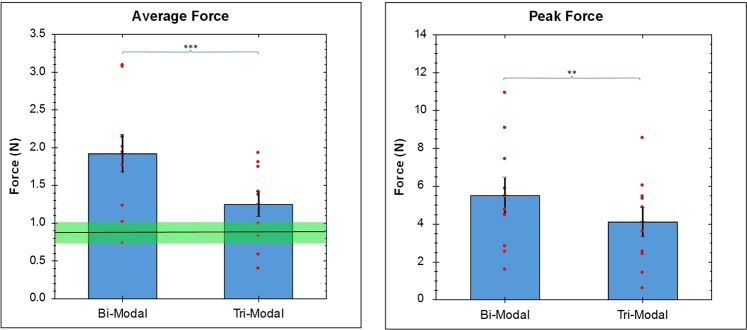
Comparison of Average and Peak Grip Force in a peg transfer study using Bi-Modal and Tri-Modal HFS. The average (and std. dev.) grip force for peg-transfer using human hands (from Fig. 1A) can be seen in green.

The results of the study based on the tri-modal HFS show (Fig. 3) a significant reduction in average grip force when the tri-modal HFS is used compared to the bi-modal kinesthetic-tactile feedback for novice subjects using the da Vinci surgical system (T-test^44^, p = 0.00039, Df = 9, T = 5.473). The same pattern followed with the peak grip force where the tri-modal feedback system showed a lower peak grip force compared to the bi-modal HFS (T-test^44^, p = 0.0049, Df = 9, T = 3.702). Figure 3 also shows the average grip force for peg-transfers using human hands (data from Fig. 1A) in green overlaid on top of the data for this study as a point of comparison.

## Discussions

Robotic surgical systems have long suffered from a lack of comprehensive haptic feedback solutions and this limitation has had significant impact on the adoption of surgical robots and in clinical outcomes^7–10,14,16^. Research in areas of haptic feedback for surgical robotics has been abundant, with recent implementations of single-modality feedback solutions showing improvements in outcomes of robotic tasks^7,10^. This investigation aimed to expand beyond single modality feedback technologies and study the potential benefits of a multi-modal feedback solution over traditional uni-modal haptic feedback systems.

The results of the experiments using the multi-modal HFS clearly highlight the benefit of multi-modal feedback over single modality feedback solutions. The observed improvements confirm that great benefits can be gained by targeting multiple mechanoreceptors in the skin and muscles simultaneously. This synergistic activation leads to a more natural sense of touch, allowing subjects to perform the task with forces nearly 50% lower than when no feedback is present (Fig. 2A).

An interesting observation from the study that is worth discussing is the larger number of faults (i.e. peg drops) in the bi-modal HFS compared to the tactile-only condition (p = 0.012). Based on the large standard error mean, it is clear that there is also significant variation among subjects. The reason for this variation is in fact due to the way the pneumatic kinesthetic feedback functions. The high pressures used for higher feedback levels makes compressing the grasper at the surgeon’s console quite challenging due to increased resistance. When this resistance is coupled with a high tactile feedback level, it appears to result in the subject suddenly relaxing their hold on the graspers, and consequently often dropping the peg. This variation is more of a learned behavior most likely caused by a lack of experience with the feedback system. Even though training with the haptic feedback system can eventually eliminate this behavior (as we observed in a series of follow up bench tests), the correct way to ultimately deal with this issue is to develop an adaptable feedback system. Such a feedback system could learn from the user’s behavior and automatically lower the pressure levels to help reduce grasper resistance and hence the number of peg drops.

Beyond the peg-transfer experiments, the results of the *ex-vivo* studies help to highlight the benefits of multi-modal HFS in more life-like surgical conditions. As expected, the bi-modal HFS reduced applied grip force in both novices and experts. However, the results also showed that such a feedback system can have a much more significant impact on the less experienced novices. In fact, novices significantly benefited from the presence of feedback with regards to the applied peak grip force. On the other hand, for surgeons, the applied peak grip force was approximately 4N, with or without feedback. That is on par with the peak grip force in novices after feedback was provided. These results help highlight the importance of training and how expert robotic surgeons have learned to compensate for the lack of feedback through the use of visual cues and overall experience. Although experienced surgeons did not reduce peak grip force when receiving feedback, the reduction in average grip force is indicative of their extensive training in robotic surgery. The improvement solely in average grip force and not peak grip force for experienced surgeons reflects their prior training in reducing applied forces and completion of tasks using solely visual cues. Whereas novice subjects lacking extensive experience with robotic systems, lack the proper skills to complete surgical tasks with or without feedback, resulting in the greater room for improvement in average grip force and peak grip force. Having said that, the average grip force data for expert surgeons under the no feedback conditions show that there is still room for improvement. That is, even for experienced surgeons, the applied average and peak forces without HFS are still high enough to induce tissue damage, as prior studies found that average forces as low as 1.25N are able to induce sites of tissue damage with number of sites increasing with greater applied force^46^, making the multi-modal HFS valuable even for expert robotic surgeons due to the evidence of average force reduction.

Even with the bi-modal kinesthetic-tactile HFS, peak grip forces which lead to significant tissue damage remain an issue. The underlying issues are the lack of training and the initial grasping action from novices that involves completely closing the console’s grasper with maximal force. While additional training can help reduce these peak forces, an alternative approach was to utilize vibrotactile feedback as a warning system which quickly activates a negative feedback response in the brain. The results clearly show that tri-modal HFS performs even more effectively than bi-modal HFS. This further confirms the initial hypothesis that controlled recruitment of additional mechanoreceptors can lead to further improvements in effectiveness of HFS.

The overlaid data from the peg-transfers performed by human hands also tells an interesting story (Fig. 3). While the sensors installed on one finger may have slightly hindered the subject’s sense of touch, the data still served as a good first level benchmark for average grip force. These data clearly highlight how close the multi-modal haptic feedback system has come to achieving what may be considered the ideal grip force. Of course, peak grip forces are still significantly higher, even with the haptic feedback system, showing that there is room for further investigation and optimization of multi-modal HFS in robotic surgery.

## Materials and Methods

### Multi-Modal HFS System Architecture

**Figure 4:**
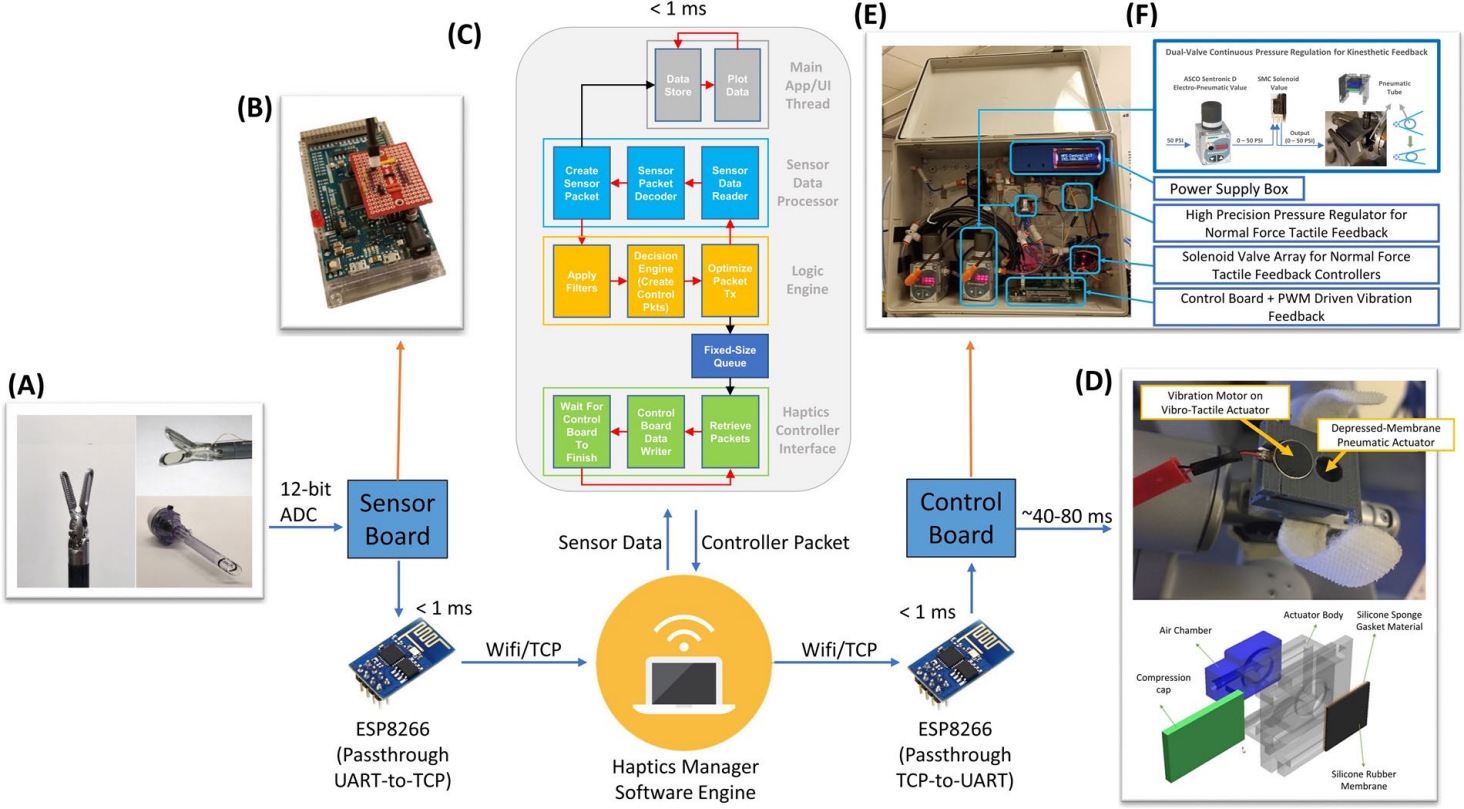
Multi-Modal HFS system architecture, Average component delays marked in milliseconds. (**A**) Sensors mounted on da Vinci instruments. Da Vinci Cadiere grasper measuring 5 mm × 14 mm shown on the left. Cadiere graspers with pressure sensors installed on the top right and 12 mm Trocar^47–49^ on the bottom right. (**B**) Sensor board. (**C**) Haptics Manager Software Architecture. (**D**) CAD model of 3D-printed depressed membrane tactile actuator and the tactile feedback actuator mounted on da Vinci controls. (**E**) Multi-Modal HFS Control hardware. (**F**) Dual-Valve pressure regulation system for use with the modified 3D-printed actuators (w/pneumatic tube for providing kinesthetic force feedback).

The multi-modal HFS architecture was designed to allow significant flexibility in the way sensor data could be mapped to activation of feedback actuators. The goal was to develop a low latency HFS that could be easily reconfigured for application in various robotic surgical tasks without requiring any reprogramming or redesign of the sensor boards and/or control boards. An overview of this system architecture can be seen in Fig. 4.

In this design, a software engine (i.e. Haptics Manager) is developed using C# and the Net framework for use as the central processing unit in the multi-modal HFS (*see Supplemental Materials Section A*). The sensor board and control board functionalities are thereby simplified. The sensor board (Fig. 4B) would thus be responsible only for transmitting raw sensor data to the Haptics Manager software, using a ESP8266 WiFi module programmed for UART-to-TCP passthrough data transmission. The control board also relies on the same communication modality for receiving control packets from the Haptics Manager software. All logic and control decisions are made in the Haptic Manager software. The software handles filtering of sensor data, deciding what actuation modality and level setting should be invoked in response to the current sensor values. A control packet is then created with the target actuation levels and sent to the control board which activates the appropriate feedback modality at the actuators mounted to the console graspers.

### Force Sensing

Because of the small size of incisions in Minimally Invasive Surgery, robotic and laparoscopic instruments are generally designed to be able to move through trocars with diameters less than 12 mm. This means that any sensor that is designed to be installed on either the end effector or on the shaft of the tool must be significantly smaller than 12 mm wide (Fig. 4A). Furthermore, the range of forces applied by different instruments can vary significantly, from 0–5N for instruments such as the da Vinci Cadiere forceps to more than 20N for the da Vinci Prograsp forceps. To meet the necessary requirements, normal force sensing is achieved through commercially available Tekscan FlexiForce piezoresistive sensors.

### Tactile Feedback

Two types of tactile feedback modalities are investigated for targeting mechanoreceptors in the skin of the fingertips. Normal force pneumatic tactile feedback was provided using a multiplexed, 5-level solenoid valve array. Controlling the activation of each solenoid valve using analog switches allows for 5 distinct pressure outputs to actuators in contact with the operator’s fingertips. This technology has previously been discussed in the literature and evaluated as an effective method for reducing grip forces in robotic surgery^26^. An alternative, depressed-membrane feedback actuator design was used to reduce the impact of sensory adaptation^31^ and allow for installation of vibration motors which provide vibrotactile feedback (Fig. 4D)^31^.

### Kinesthetic Force Feedback

Kinesthesia and proprioception are often interchangeably used in the literature to refer to the awareness of the position and movement of the limbs and the muscles. Proprioception is more strongly linked to the feedback mechanisms within the neuromuscular system, as it involves the process of sending information about the movement of the body to the brain in order to make proper adjustments to muscle movements. Proprioceptive Feedback, or as it is more commonly known, Kinesthetic Force Feedback (KFF), is therefore designed to trigger activation of mechanoreceptors in the muscles, particularly the Golgi tendon organ (GTO). The GTO is responsible for measuring tension in the muscles and sending this sensory information to the brain to help create a sense of resistance^50^.

Conventional implementations of KFF often rely on motors installed on the joints of the robotic controls to provide feedback. This approach, particularly for grasping applications, can be susceptible to a jerking phenomenon which results from the interaction of KFF with human reflex responses (*see Supplemental Materials Section B*). A pneumatic kinesthetic force feedback system was developed as an alternative, aiming to provide a more natural sense of kinesthesia. This solution relied on the placement of a pneumatic tube between the graspers of the master console.

The increase in air pressure (0–19 PSI) inside the tube would lead to constriction of the grasper’s ability to close, hence resisting the grasping action. The higher the pressure inside the tube, the more the grasper would resist being closed, indicative of stronger kinesthetic feedback.

One of the disadvantages of the motor-based kinesthetic feedback design is its dependency on modifications to the robotic console. The pneumatic feedback was instead designed as an add-on solution with compatibility in mind. The 3D printed pneumatic actuators, which already supported vibration feedback, were modified even further to allow installation of the kinesthetic feedback system directly on the same actuator (Fig. 4F).

In order to control the air pressure for this kinesthetic feedback system, it was necessary to investigate an alternative pressure regulation system compared to the one in use for the normal force tactile feedback. The reason for this change from the quantized levels of our previous tactile feedback system^46^ was the lack of scalability. With the utilization of the multiplexed solenoid valve array, each additional pressure level, for each actuator, required one additional solenoid valve. For this reason, providing a pressure control system that would simulate continuous pressure regulation would require an extremely large number of solenoid valves, a design which, due to cost and complexity of the system, would not be practical. A more compact pressure regulation system with a high number of pressure levels (i.e. simulating continuous pressure regulation), was critical for two reasons: (1) large pressure changes can reduce user performance, particularly in a kinesthetic feedback where sudden changes in feedback can result in the user dropping the peg, and (2) a user-based, adaptive feedback requires pressure levels to be variable and changeable electronically.

Previous research had investigated the possibility of utilizing electro-pneumatic pressure regulators. However, these systems generally have slow response times and produce significant vibration during pressure changes^46^. To resolve these issues, a dual-valve continuous pressure regulation system was developed that would provide continuous pressure regulation with low latency.

The dual-valve pressure regulation technology relied on an Asco Sentronic D electro-pneumatic valve placed in series with a SMC solenoid valve. The Sentronic D valve utilizes a direct acting proportional coil to control output pressure. The benefit of this type of electro-pneumatic valve is its stable pressure output. The stable response, however, comes at the expense of system response times. System response time can be reduced, but reducing the response time comes at the expense of large pressure overshoots when pressure levels are being changed. The Sentronic D’s output pressure is controlled by providing a voltage of 0–10 V to its analog control pin.

This large pressure overshoot could not be allowed to reach the pneumatic tubing. To resolve this issue, a dual-input solenoid valve was placed in series after the Sentronic D valve (Fig. 4F). The solenoid valve, when in the off position, would allow air flow from the first input to its output. When turned on, the solenoid valve would allow air flow from the second input to its output. The haptic feedback controller was then programmed to change the solenoid valve to its on position right before changing the pressure of the Sentronic D valve. By feeding the output of the solenoid valve back to its second input port, the system would maintain the output pressure until the Sentronic D’s output pressure had stabilized. At that time, the solenoid valve would be turned off, and the new pressure would flow to the actuators.

The Sentronic D valve has a built-in controller which can be programmed using Asco’s data acquisition software. By modifying various control parameters of the valve, a stable pressure output was achieved while also reducing the total response time of the dual-value system to <70 ms (Average: 65 ms, Std. Dev.: 7.5 ms, Max: 67 ms, Min: 50 ms).

### Grip-Force Reduction Studies: Experimental Methods for Evaluation of Hybrid HFS

All methods were previously approved by the Institutional Review Board at UCLA and carried out in accordance with relevant university guidelines and regulations. Per approved IRB protocol, informed consent was obtained from all subjects.

### Evaluation of Bi-Modal Pneumatic Kinesthetic-Tactile HFS

**Figure 5:**
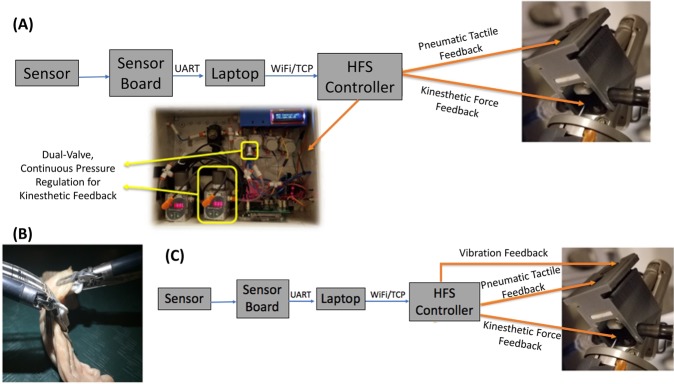
Grip Force reduction studies. (**A**) Control System for a Bi-Modal Pneumatic Kinesthetic Tactile HFS. (**B**) HFS *Ex-Vivo* porcine large intestine handled using da Vinci IS1200 Cadiere forceps. (**C**) Control system for a Tri-Modal HFS.

These experiments targeted the evaluation of a hybrid kinesthetic-tactile HFS using the pneumatic implementation of KFF on the da Vinci IS 1200 surgical system. The control system for the pneumatic kinesthetic-tactile hybrid HFS was implemented by configuring the logic engine of the Haptics Manager software to utilize the dual-value continuous pressure regulators as a means of changing the pressure in the pneumatic tube responsible for providing kinesthetic force feedback (Fig. 5A).

A total of 15 novice subjects with little to no experience with robotic surgery were recruited to perform single handed peg-transfer tasks using a da Vinci IS1200 surgical system. Subjects were given a 2-minute training period prior to the start of the study to familiarize themselves with the robotic system. This training period was sufficient in most cases since the use of clutch, camera and most other complex da Vinci operations were not allowed. In order to eliminate any bias toward either hand, nearly half (7) of the subjects received feedback on the right hand while the remaining subjects (8) received feedback on the left hand. All subjects were right handed. Each subject was asked to perform four peg transfers per trial. To eliminate any bias towards the position in which pegs may be dropped, if the subject dropped the peg, the proctor would quickly reset the peg to its original position.

For this study, a FlexiForce A201 sensor was installed on da Vinci Fenestrated Bipolar forceps. Each subject performed the peg transfer tasks four separate times as part of four trials, with each trial performed under different feedback conditions: (1) No Feedback (2) Normal Force Tactile Feedback (3) Pneumatic Kinesthetic Force Feedback (4) Hybrid Kinesthetic-Tactile Feedback.

During the trial, the number of faults (i.e. number of times the subject dropped the peg), time-to-completion, and the grip force were recorded. Statistical analysis of average grip force was performed using Repeated Measures ANOVA^42^ after a Log2 transform was used to meet the normality assumption. Repeated Measures ANOVA^42^ was also used for analysis of peak grip force. Statistical analysis of the number of faults was conducted using Ordinal Repeated Measures ANOVA. Follow up post-hoc analysis using Tukey correction^43^ were performed when p-value was less than 0.05.

### *Ex-Vivo* Evaluation of Bi-Modal Kinesthetic-Tactile HFS

In order to evaluate the bi-modal kinesthetic-tactile HFS under conditions more representative of real-world surgical applications, an *ex-vivo* study was designed. Previous *in-vivo* work on tactile feedback systems have shown that there is a clear correlation between applied grip force and the number of damaged tissue sites^46^. Since the correlation of applied grip force with tissue damage already exists, it is therefore possible to rely on *ex-vivo* experiments for evaluation of the kinesthetic-tactile HFS.

These experiments relied on the same control system as the previous pneumatic kinesthetic-tactile HFS. FlexiForce A201 sensors were installed on two da Vinci Cadiere forceps. Feedback actuators were installed on both left and right controls of the da Vinci system console. Only one hand however received feedback throughout the trial. This experimental design choice allowed one hand to act as a control for the other. To eliminate any bias toward either hand, half of the subjects performed the task with feedback on one hand and half with feedback on the other.

Subjects were asked to run a porcine bowel, approximately 30 cm in length (Fig. 5B). Each subject performed the task only once. For this investigation, two groups of subjects were recruited. The first study was performed by recruiting 10 novice subjects with little to no experience with robotic surgery. The second group of subjects were expert robotic surgeons recruited from Ronald Reagan Hospital at the University of California, Los Angeles. A total of 8 expert surgeons were recruited for participation in this study.

For all subjects, applied grip force was recorded throughout the study. For expert subjects, the number of times the surgeon dropped the tissue with either hand was also recorded to determine any impact of the hybrid HFS on proper handling of the tissue. This parameter (number of faults) was not recorded and analyzed for novices. This experimental design choice was made because of the difficulty that many novice subjects experienced picking up and handling the bowel tissue. This lack of experience and the non-homogeneity of the tissue made this parameter too variable in novices to be valuable without requiring a very large number of subjects to be recruited. For novice subjects, the study controlled for utilization of the clutch^30^ by prohibiting subjects from using the clutch operation on the da Vinci. The proctor instead adjusted the controls such that no visual-perceptual mismatch^51^ was present and subjects could perform the tasks without requiring access to the clutch and camera operations.

Statistical analysis for any one of the measured metrics (ex. force, faults, etc.) was performed using a standard student’s paired t-test^44^ when normality assumption was met, and a non-parametric, paired Wilcoxon Signed Rank test^45^ when the data were not normally distributed.

### Evaluation of a Tri-Modal HFS

A tri-modal haptic feedback system was utilized to provide a third modality of feedback when applied forces moved beyond a certain threshold. Building upon previous understanding of the effectiveness of vibrotactile feedback as a warning system^30^, these experiments aimed to determine the potential benefits of a tri-modal HFS over the previously tested bi-modal kinesthetic-tactile feedback system in reducing average and peak grip forces in robotic minimally invasive surgery tasks.

The control system for a tri-modal feedback system was implemented by configuring the logic engine of the Haptics Manager software (Fig. 5C).

A total of 10 subjects were recruited to perform a two-handed peg transfer task using a da Vinci IS1200 surgical system. Two Cadiere forceps were installed with FlexiForce A201 normal force sensors. Subjects received bi-modal kinesthetic-tactile feedback on one hand and tri-modal feedback on the other. The tri-modal feedback rules were the same as the bi-modal ones with the difference that vibration feedback was provided beyond level 3 of normal force tactile feedback (2N). A more intense vibration was provided beyond level 4 of normal force feedback (3N). To eliminate bias towards either hand, the hand receiving tri-modal feedback was switched for half of the subjects.

For all subjects, the study controlled for utilization of the clutch by prohibiting subjects from using the clutch operation on the da Vinci. The proctor instead adjusted the controls such that no visual-perceptual mismatch was present and subjects could perform the tasks without requiring access to the clutch operation.

Subjects were asked to perform four peg transfers. In each transfer, the subject would pick up the peg on one side of the field with the arm closest to the peg, pass it to the other arm, and then place it back down on the other side of the field. The applied grip force and the number of peg drops with each hand was recorded throughout the trial. Statistical analysis was performed using a standard Student’s paired t-test^44^ when normality assumption was met and a Wilcoxon Signed Rank test^45^ when the data were not normally distributed.

To allow a qualitative comparison of the system with the normal sensory feedback system of the human hand, 6 subjects were asked to perform the same peg-transfer with their own hands (thumb and index finger only) with the sensors used in the study installed on their index fingers. The force values during 10 consecutive peg two-handed peg transfers were used to calculate the average grip force when performing this task using normal human senses.

## Supplementary information

Multi-Modal HFS Manuscript (Supplemental)
