# Supplementary material for: Multi-Modal Haptic Feedback for Grip Force Reduction in Robotic Surgery
Source: Sci Rep. 2019 Mar 21;9:5016. doi: 10.1038/s41598-019-40821-1 (PMC6428814; doi:10.1038/s41598-019-40821-1)
Supplement: Supplementary file 1 — Multi-Modal HFS Manuscript (Supplemental) [file 41598_2019_40821_MOESM1_ESM.pdf]

# Multi-Modal Haptic Feedback for Grip Force Reduction in Robotic Surgery

Ahmad Abiri[1,2*], Jake Pensa[1,2], Anna Tao[1], Ji Ma[2], Yen-Yi Juo[3], Syed J. Askari[1,2], James Bisley[4], Jacob Rosen[2], Erik P. Dutson[1,3] and Warren S. Grundfest[1,2]

[1]UCLA Center for Advanced Surgical and Interventional Technology (CASIT), [2]UCLA Henry Samueli School of Engineering and Applied Science, [3]UCLA Department of Surgery, [4]UCLA Department of Neurobiology, *Ahmad Abiri aabiri@ucla.edu

## Supplemental Materials

### A. HFS Control System Software

The Haptics Manager software serves as the primary filter and control component of the multi-modal haptic feedback system. The software is a C# application developed using Microsoft's .Net framework. In the most abstract way, the software is designed to receive sensor data in a particular packet format and generate a controller packet in response.

Fig. *4*C illustrates the Haptics Manager software architecture. The Haptics Manager provides four major functions:

1. Decode & Filter Raw Sensor Data (Sensor Data Processor)
2. Generate Control Board Packets (Logic Engine)
3. Transmit Data to Control Board (Haptics Controller Interface)
4. Plot Data and Store Sensor Data in Real-Time (Main UI)

The Haptics Manager software architecture was designed with data processing latency in mind. An asynchronous, multi-threaded architecture with fixed-size processing queues was used in the design of Haptics Manager software architecture. In Fig. 4C, transition of data between processing units within the same thread can be seen in red, while cross-thread operations can be observed in black. The purpose of the fixed-size queue is to ensure that any slowdowns in handling the controller packets, whether it'd be related to communication, or to mechanical delays in the actuators, would have no impact on data processing. Under such conditions, the queue can become full and then begin to lose older items as new items are inserted. When the controller thread begins responding again, a few packets may have been skipped, but the system quickly begins to respond to the most recent data, rather than spending time processing previous requests that may no longer be relevant. This approach allows the system to quickly recover from any unexpected delays within any component. The same benefits also apply to the data storage and user interface updates which suffer slowdowns particularly when the computer's hard disk or graphics card are busy with another task. The resulting software has an average processing latency < 1ms (tested on Intel Core i3-5005U processor).

### B. Conventional Kinesthetic Force Feedback

KFF technologies which aim to simulate this effect traditionally rely on motors placed at the hinges of robotic controls to increase the resistance of the joint to movement. In order to provide KFF at the surgeon's fingertips for tasks involving grasping, a motor installed on the hinge of RAVEN surgical system's robotic console was used (Supplementary Fig. S6A-C).

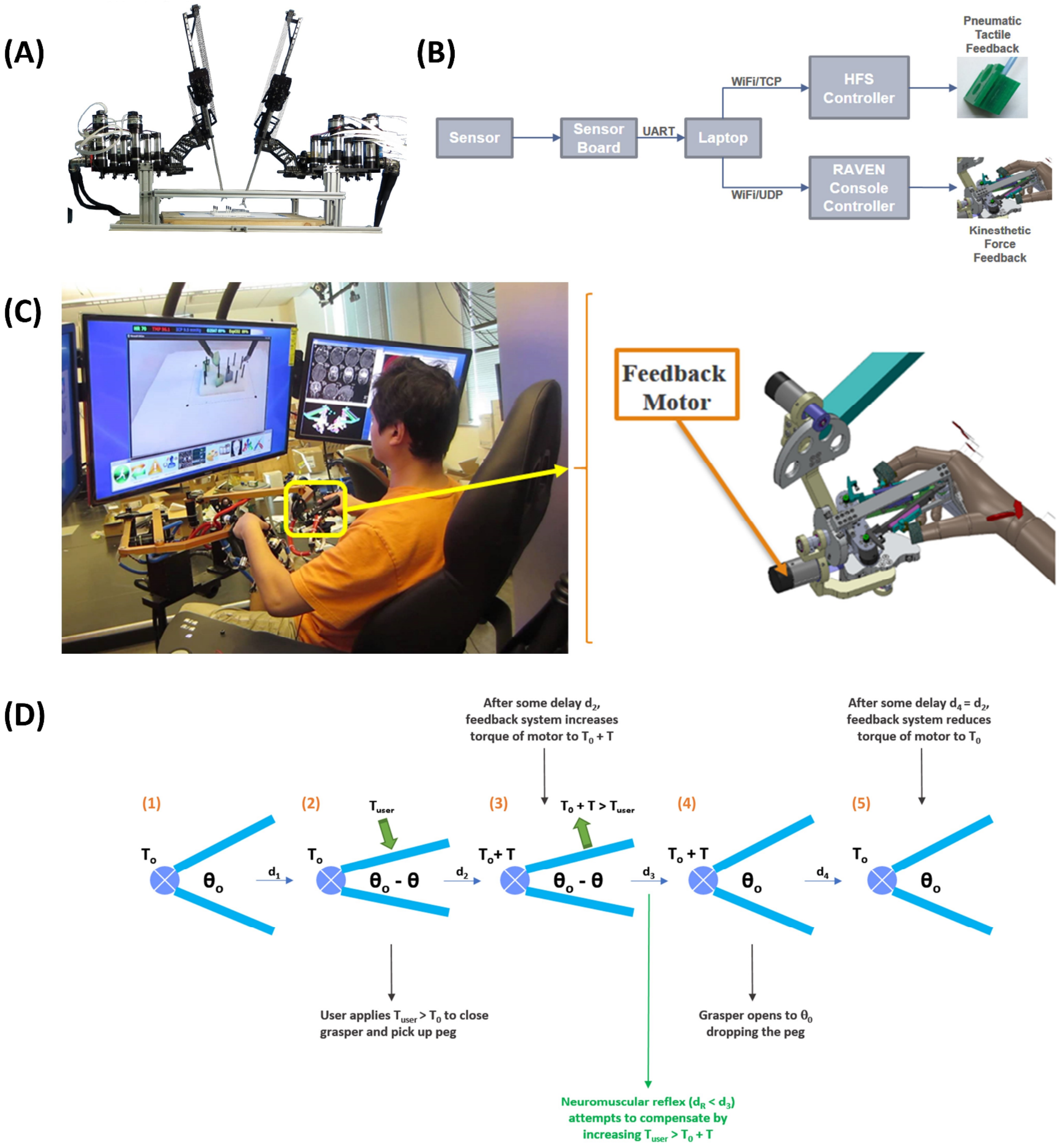


**Supplementary Fig. S6. Implementation of Kinesthetic Force Feedback on RAVEN surgical system. (A)** RAVEN Surgical Robot. **(B)** Control System of the Kinesthetic-Tactile Hybrid HFS utilizing the RAVEN console motor for Kinesthetic Force Feedback. **(C)** RAVEN surgical console with the feedback motor installed on the RAVEN surgical console controls. **(D)** Series of events leading to subject dropping the peg when Kinesthetic Feedback is provided on the RAVEN: (1) Relaxed RAVEN joystick/grasper, (2) User presses and closes the grasper, (3) Force from motor increases in response to signal from sensors, (4) Higher forces overcome user's applied muscle force, opening grasper to $\theta_o$, (5) Force on the motors is reduced since sensors aren't detecting high forces.

A UDP server was installed on the RAVEN console's control software which received a value between 0-99, indicating the amount of power that would be provided to the feedback motor. This would in turn control the resistance the motor would provide when the user attempted to close the grasper. A UDP client was also built into the Haptics Manager software which would allow the Logic Engine to send kinesthetic feedback levels to the RAVEN console. This

approach allowed the haptic feedback system to provide kinesthetic feedback in response to increasing grip forces detected by the sensors installed on the graspers of the RAVEN surgical system.

## Evaluation of Bi-ModaL Conventional Kinesthetic-Tactile HFS

These experiments targeted the evaluation of a hybrid kinesthetic-tactile HFS using conventional implementation of KFF on the RAVEN surgical system. The control system of the hybrid kinesthetic-tactile HFS was implemented by configuring the Haptics Manager software engine to send kinesthetic force levels to an external kinesthetic feedback control software running on the RAVEN surgical console through UDP (Supplementary Fig. **S6**B).

A total of 17 novice subject with limited or no experience with robotic surgery were recruited from the general population at the University of California, Los Angeles. Subjects were given a 5-minute training period prior to the start of the study to familiarize themselves with the RAVEN surgical system. The study utilized a repeated measures design in which each user performed a single-handed peg transfer task, as part of seven trials under different feedback conditions: (1) No Feedback (2) Kinesthetic Force Feedback (3) Normal Force Tactile Feedback (4) Hybrid Kinesthetic-Tactile Feedback. Each trial involved two, single-handed peg transfer tasks adopted from the standard peg transfer test in the Fundamentals of Laparoscopic Surgery (FLS) education module developed by the Society of American Gastrointestinal Endoscopic Surgeons (SAGES)[S47]. For recording the applied force to the peg, FlexiForce A201 sensors were installed on the RAVEN surgical robot's forceps.

The seven trials consisted of four No Feedback trials separating three feedback trials. The three feedback trials were randomized to eliminate any training bias toward either feedback modality. The four no feedback trials (NF1 through NF4) were also spread out across the session (in between the feedback trials) in order to observe any possible learning effect over the duration of the seven trials. Subjects were also given a 5-minute training period prior to the experimental trials to familiarize themselves with the controls of the RAVEN robotic system. During the trial, to eliminate any bias toward the position which a subject may drop the peg, if the subject dropped the peg, the proctor would quickly reset the peg to its original position.

During the trial, the number of faults (number of times the subject dropped the peg), time-to-completion. and applied grip force were recorded. Statistical analysis of average grip-force was later performed using Rank Transformed Repeated Measures ANOVA due to non-normality of the data. Statistical analysis of time-to-completion was performed using Repeated Measures ANOVA following a Log2 transformation to achieve normality. For analysis of the number of faults, Ordinal Repeated Measures ANOVA was used. Follow up post-hoc analysis using Tukey correction were performed when p-value was less than 0.05.

## Results: Bi-ModaL Conventional Kinesthetic-Tactile HFS

The results of the study for the number of faults, time-to-completion and average grip-force are shown in Supplementary Fig. S7. The data show no significant difference between any of the

groups with regards to the number of peg drops (ANOVA, $p = 0.534$) and time-to-completion of the task (ANOVA, $p = 0.119$).

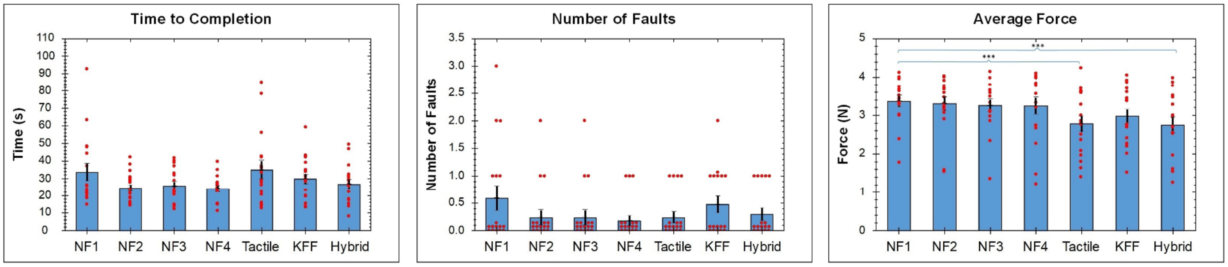


**Supplementary Fig. S7. Evaluation of performance under different feedback conditions using RAVEN surgical system.** Comparison of Time-to-Completion, Number of Faults (Number of Peg Drops), and Average Grip-Force under different feedback conditions using the RAVEN surgical system.

The average grip force was significantly lower when Hybrid HFS was used compared to all No Feedback (NF) conditions (Tukey, $p = 0.00069$ for Hybrid vs. NF1). The same trend was present for Tactile Feedback (Tukey, $p = 0.0022$ for Tactile vs. NF1). No significant difference was observed between the kinesthetic feedback and any of the No Feedback conditions. Additionally, there was no significant difference between the four No Feedback conditions, indicating that there is no reduction in grip force, number of faults, and time-to-completion resulting from learning behavior.

As expected, normal force tactile feedback leads to reduction of grip forces compared to the No Feedback conditions. This is consistent with findings of previous work in this area[41]. However, the results of this study did not meet the original expectations for provision of kinesthetic force feedback. In fact, there did not appear to be any benefit from the kinesthetic force feedback at all. The results also seem to point out that any benefit from the hybrid kinesthetic-tactile HFS was in fact only caused by the pneumatic normal force tactile feedback, and not the kinesthetic force feedback.

While the indication that kinesthetic-force feedback was ineffective in improving outcomes was initially surprising, further analysis of observations from the study shed light on the underlying cause.

The data in Supplementary Fig. S7 show that when kinesthetic feedback was activated, the average grip force was slightly lower compared to the No Feedback condition. While this difference was not significant, it may indicate that the issue causing the ineffectiveness of kinesthetic force feedback may result from a more complex underlying problem, rather than the kinesthetic feedback modality itself.

The ineffectiveness of kinesthetic force feedback was in fact caused by the subject's neuromuscular reflex to a series of events which would otherwise result in the subject dropping the peg. Supplementary Fig. **S6**D describes these events in five steps. At initial time, the feedback motor applies a normal force $F_0$ to the subject's hand for any angle larger than $\theta_o$,

where $\theta_o$ is the maximum opening angle of the grasper. This initial condition (1) is followed by (2) the user applying some addition force to the grasper to close it, after which the closed grasper comes in contact with a peg, causing the feedback system to response by (3) increasing the feedback motor power, hence applying a higher force ($F_0 + F$) to the subject's hand, after which (4) the feedback motor now overcomes the force applied by the subject's fingers and returns to its original maximum open angle of $\theta_o$. By this time, the robotic arms have dropped the peg, hence telling the system to (5) reduce the feedback motor power, returning the system to its initial condition. Supplementary Fig. **S6**D shows the delays between each event as $d_1$ through $d_4$. According to the events described above, the subject should drop the peg every time he/she attempts to pick it up, because if the human neuromuscular reflexes are longer than $d_3$, then there is no way the subject has enough time to increase the force applied by his/her finger in time to prevent the grasper from opening all the way to $\theta_o$ and releasing the peg. However, the human neuromuscular response is more complicated. In fact, in some subjects, the response is faster, allowing the subject to prevent the peg drop. In other subjects, the brain quickly learns and predicts this event, thereby forcing the fingers to apply a higher initial load, one that would not be overcome by $F_0 + F$, but this extra load comes at the expense of excessive force applied to the peg.

This unexpected phenomenon arises from two fundamental problems in the kinesthetic force feedback system. The first is that the feedback delay ($d_2$ and $d_4$), that is, the time it takes for values from the sensor to impact the force of the feedback motor is longer than $d_3$. If we were to assume that this feedback delay was zero, in such a case, the grasper is opening during transition from (3) to (4), the applied force on the sensors would be reduced as well, hence reducing the applied feedback force, which in turn, at some point, would reach a level low enough that it would no longer overcome the subject's muscular tension. Of course, such a system with zero delay is not feasible and further reduction of delays would not guarantee a complete resolution of this problem either. The reason is that there is a second, more fundamental issue with this implementation of kinesthetic force feedback. That is, this form of kinesthetic force feedback does not mimic the behavior of real-world objects and how human hands interact with them. As opposed to the grasper which tries to force its way back to $\theta_o$, a real-world object does not stretch out beyond its original size and shape when it is compressed. It simply resists the compression further. Therefore, any HFS that attempts to mimic real-world kinesthetic feedback must not rely on a spring-like response for providing feedback, but rather replicate that sense of resistance.

These results and observations were the fundamental motivation behind the investigation of kinesthetic force feedback using the pneumatic kinesthetic-tactile HFS.
